# ETHICAL CONSIDERATION IN HOME MONITORING ASSISTIVE ROBOT FOR COGNITIVE IMPAIRMENT: A SCOPING REVIEW

**DOI:** 10.1093/geroni/igad104.3307

**Published:** 2023-12-21

**Authors:** Jing Wang, Dain LaRoche, Momotaz Begum, Cassidy Choquette, Xueting Tang, Sajay Arthanat

**Affiliations:** The University of New Hampshire, Durham, New Hampshire, United States; The University of New Hampshire, Durham, New Hampshire, United States; The University of New Hampshire, Durham, New Hampshire, United States; The University of New Hampshire, Durham, New Hampshire, United States; Fudan University, Shanghai, Shanghai, China (People’s Republic); The University of New Hampshire, Durham, New Hampshire, United States

## Abstract

As the aging population continues to grow, the demand for innovative technologies to support individuals with cognitive impairment in their daily lives has risen significantly. Home monitoring assistive robots (HMAR) have emerged as a promising solution, offering assistance with tasks, scheduling and safety monitoring. This scoping review aims to explore and synthesize the ethical considerations surrounding the use of HMARs for persons with cognitive impairment. We adopted the Arksey and O’Malley framework, encompassing five stages: identifying the research question, identifying relevant studies, selecting studies, charting the data, and summarizing and reporting results. A comprehensive search strategy was employed across electronic databases, including PubMed, PsycINFO, Scopus, and CINAHL, using search terms derived from titles, abstracts, and keywords identified in key publications and previous reviews relevant to the scope of this review. The synthesis of the 25 included literature highlights several key ethical dimensions related to HMARs for individuals with cognitive impairment, including autonomy and decision-making, privacy and surveillance, family dynamics and dyadic relationships, human-robot trust, cultural considerations, and equitable access to technology. The review underscores the complexity of ethical dilemmas posed by HMARs, including issues of informed consent, data security, power differentiation between persons with cognitive impairment and their caregivers, and disparities in technology access. The findings of this scoping review contribute to the ongoing discourse on the ethical implications of utilizing HMARs for cognitive impairment and provide insights for the development of responsible and inclusive technological solutions.

